# Social Cognitive Predictors of Health Promotion Self-Efficacy Among Older Adults During the COVID-19 Pandemic

**DOI:** 10.1177/08901171241256703

**Published:** 2024-05-30

**Authors:** Michelle C. Yang, Gurkaran Singh, Brodie M. Sakakibara

**Affiliations:** 1 Graduate Programs in Rehabilitation Sciences, 12358 University of British Columbia, Vancouver, BC, Canada; 2 Centre for Chronic Disease Prevention and Management, 97950 University of British Columbia, Kelowna, BC, Canada; 3 Department of Occupational Science and Occupational Therapy, 12358 University of British Columbia, Vancouver, BC, Canada

**Keywords:** older adults, age specific, specific populations, health promotion self-efficacy, COVID-19, cross-sectional study, quantitative research, performance accomplishment, vicarious learning, verbal persuasion, affective state

## Abstract

**Purpose:**

To examine the relative importance of social cognitive predictors (ie, performance accomplishment, vicarious learning, verbal persuasion, affective state) on health promotion self-efficacy among older adults during COVID-19.

**Design:**

Cross-sectional.

**Setting:**

Data collected online from participants in British Columbia (BC), Canada.

**Subjects:**

Seventy-five adults (n = 75) aged ≥65 years.

**Measures:**

Health promotion self-efficacy was measured using the Self-Rated Abilities for Health Practices Scale. Performance accomplishment was assessed using the health directed behavior subscale of the Health Education Impact Questionnaire; vicarious learning was measured using the positive social interaction subscale of the Medical Outcomes Survey - Social Support Scale (MOS-SSS); verbal persuasion was assessed using the informational support subscale from the MOS-SSS; and affective state was assessed using the depression subscale from the Depression Anxiety Stress Scale (DASS-21).

**Analysis:**

Multiple linear regression was used to investigate the relative importance of each social cognitive predictor on self-efficacy, after controlling for age.

**Results:**

Our analyses revealed statistically significant associations between self-efficacy and performance accomplishment (health-directed behavior; β = .20), verbal persuasion (informational support; β = .41), and affective state (depressive symptoms; β = −.44) at *P* < .05. Vicarious learning (β = −.15) did not significantly predict self-efficacy. The model was statistically significant (*P* < .001) explaining 43% of the self-efficacy variance.

**Conclusion:**

Performance accomplishment experiences, verbal persuasion strategies, and affective states may be the target of interventions to modify health promotion self-efficacy among older adults, in environments that require physical and social distancing.

## Purpose

The COVID-19 pandemic has drastically impacted numerous lives at a global scale.^1^ Subsets of the general population, namely older adults, have experienced increased hardships not only because of the virus itself, but due to protective measures to mitigate its spread. These measures include factors such as the use of face masks, social distancing practices, and social gathering restrictions.^2^ These measures can be especially impactful among older adults as they can result in adverse outcomes such as physical inactivity, social isolation, and sleep disturbance, which have been identified as outcomes that reduce wellbeing and increase risk of major pre-existing health conditions, cognitive impairment, and death among older adults across several pre-pandemic studies.^3-7^ Furthermore, a study found that a significant number of individuals living with non-communicable, chronic diseases (eg, cardiovascular disease or diabetes) during the COVID-19 pandemic reported worsening physical health and significant increases in weight, tobacco consumption, and mental health symptoms compared to pre-pandemic times.^8^ Moreover, multiple studies have reported negative outcomes in health behaviours among older adults because of the pandemic, including significant decreases in moderate and vigorous physical activity and increases in sedentary time.^3^ Thus, there is an importance to better understand how older individuals supported their own health during the COVID-19 pandemic.

Multiple factors can motivate an individual to adopt healthy behaviours. One factor that may plausibly support individuals in managing their health behaviours during times like the COVID-19 pandemic is health promotion self-efficacy.^1^ Health promotion self-efficacy is the belief individuals have in their ability to perform desired actions to improve their health, and has been shown to be an important predictor of actual health behaviours.^9,10^ For example, a study among individuals with multiple sclerosis indicated that health self-management and health promotion self-efficacy improved healthy eating behaviours and found that age was a significantly influential factor among older adult participants being able to better cope with health problems over time.^11^ Furthermore, a study on factors impacting healthy aging among older adults in China found that factors such as health promotion self-efficacy and family networks had a strong impact on healthy ageing.^12^ These finding are likely a result of self-efficacy being a psychological factor that improves prior to modifying subsequent health behaviours.^13^ Thus, health promotion self-efficacy has important implications for health self-management behaviours, which may make it an important factor to consider in the context of COVID-19.^14^

Bandura’s Social Cognitive Theory (SCT) explains that self-efficacy may be modified via 4 sources of information: performance accomplishment, verbal persuasion, vicarious learning, and affective state,^15^ and is context specific. While these sources of information have been clearly defined in the literature to influence self-efficacy, there is little information on which variables have the most influence on an individual’s health promotion self-efficacy in the context of social and physical restrictions, such as those in place during the COVID-19 pandemic. Furthermore, as previous research on predictors of SCT have been done before the implementation of social distancing, it is important to consider whether these 4 sources of information for health-promotion self-efficacy are valid in pandemic times. In this study, we examine the relative importance of social cognitive predictors on health promotion self-efficacy among community-living adults, aged >65 years during the COVID-19 pandemic. It is hypothesized that among community-living adults aged 65 years or older that the 4 sources of information from SCT (performance accomplishment, verbal persuasion, vicarious learning, and affective state) will be statistically significant (*P* < .05) independent predictors of health promotion self-efficacy after controlling for age.

## Methods

### Design

This cross-sectional study is a secondary analysis of baseline data from a larger interventional study, that investigated the feasibility of a COVID-19 chronic disease self-management support intervention among older individuals.^16^ Ethical approval was obtained from the Behavioural Research Ethics Board (H20-01368) at the University of British Columbia (UBC). Reporting is in accordance with the Strengthening the Reporting of Observational Studies in Epidemiology (STROBE) checklist (Supplemental Material 1).^17^

### Sample

Individuals were recruited via convenience sampling from British Columbia (BC), Canada via social media, e-blasts to patient advocacy groups and local community organizations, and a study webpage. To be included for study, individuals needed to: (1) be 65 years or older; (2) be living in the community; (3) communicate in English, and (4) provide informed consent. Individuals were excluded if they were not medically stable and/or had a cognitive impairment. In total, seventy-five participants (n = 75) were recruited in the larger study, the data of which are included in this secondary analysis.

### Measures

Participants attended a 90-minute online data collection session with a trained outcomes assessor. Participants completed a socio-demographic information form along with the following questionnaires that captured the study variables of interest.

Dependent variable: Health promotion self-efficacy was measured using the 28-item Self-Rated Abilities for Health Practices (SRAHP) Scale, which has high test-retest reliability (Pearson Correlations = .70) and good validity (General Self-Efficacy scale r = .43).^18^ All questions are measured on a 5-point Likert scale ranging from zero *(“not at all”)* to 4 *(“completely”)*.^18^ Scores from each question are totalled (total score = 112) with higher overall scores indicating higher health promotion self-efficacy (ie, nutrition, exercise, psychological wellbeing, and responsible health practices).^18^

Independent variables (social cognitive sources of information): i) Performance accomplishment was measured using the Health Directed Behaviour subscale of the 40-item Health Education Impact Questionnaire (heiQ),^19^ with higher scores indicating greater performance accomplishment; ii) vicarious learning was measured using the positive social interaction subscale of the Medical Outcomes Study: Social Support Survey (MOS-SSS),^20^ with higher scores indicating greater vicarious learning; iii) verbal persuasion was measured using the informational support subscale of the MOS-SSS,^20^ with higher scores indicating greater verbal persuasion; and iv) affective state was measured using the depressive symptoms subscale of the 21-item Depression, Anxiety and Stress Scale (DASS-21), with higher scores indicating lower affective state.^21^

### Analysis

Descriptive statistics were calculated for all variables. Correlations were derived between self-efficacy and all independent variables. Small, medium, and large correlations were defined as being .10, .30, and .5, respectively.^22^ All regression assumptions were tested (ie, collinearity, homoscedasticity, linearity, and normally distributed residuals) and met before proceeding with our regression analysis. Multiple linear regression analyses were then conducted using SPSS-27 to examine the associations between the 4 sources of information (performance accomplishment, vicarious learning, verbal persuasion, and affective state) and health-promotion self-efficacy. Beta coefficients were used to determine the relative importance of each of the social cognitive predictors, with higher coefficients indicating a stronger association with health promotion self-efficacy. Age was first entered into the regression model (Model 1), followed by the 4 sources of information (Model 2).

## Results

### Descriptive Statistics

Study participants (n = 75) had a mean age of 72.4 years and 58.7% were female (Table 1). Fifty-seven participants (76%) were Caucasian, and 46 (61%) reported living with 3 or more chronic conditions.

**Table 1.** Sample Characteristics (N = 75).

Demographic	Value	μ±SD or n (%)
Age	Years	72.4 ± 5.8
Sex	Male	31 (41.3)
	Female	44 (58.7)
Education	Post-secondary	67 (89.3)
Number of chronic conditions	1	11 (14.7)
	2	13 (17.3)
	≥3	46 (61.3)
Employment status	Employed	14 (18.7)
	Unemployed/Retired	61 (81.3)
Geographic region	Urban	43 (57.3)
	Suburban/Rural	32 (42.7)
Race/Ethnicity	White or caucasian	57 (76.0)
Outcome variables	Health promotion self-efficacy/112	87.4 ± 13.3
	Performance accomplishment/5	4.1 ± 0.9
	Vicarious learning/4	3.7 ± 1.1
	Verbal persuasion/4	3.6 ± 1.0
	Affective state/21	5.8 ± 7.1

### Correlation Analyses

Performance accomplishment, vicarious learning, and verbal persuasion as measured by health directed behaviour, positive social interaction, informational support subscales, respectively, were each shown to have a positive, medium correlation with self-efficacy (*P* < .05). Affective state, as measured by the depressive symptoms’ subscale on the DASS-21, had a negative, moderate correlation with self-efficacy (*P* < .05) (Table 2).

**Table 2.** Correlation and Regression Modeling to Identify Predictors of Health Promotion Self-Efficacy (N = 75).

	r	Model 1				Model 2			
Variable		b	95% CI (b)	SE	β	b	95% CI (b)	SE	β
Constant		77.96	39.06, 116.86	19.52		46.38	10.53, 82.22	17.97	
Age		.13	−.41, .67	.27	.06	.28	−.14, .70	.21	.12
Performance accomplishment	**.35**					3.08	.21, 5.95	1.44	.20
Vicarious learning	**.43**					−1.93	−5.76, 1.90	1.92	−.15
Verbal persuasion	**.51**					5.55	1.61, 9.50	1.98	.41
Affective state	**−.57**					−.82	−1.20, −.45	.19	−.44
Adjusted *R*^*2*^		1%				43%			

**Bold** indicates significance at alpha of .05 (two-tailed).

### Predictors of Health Promotion Self-Efficacy

Collinearity, or strong correlation between 2 independent variables, was determined by a variance inflation factor of 10 or greater or an intercorrelation of .70 or greater between independent continuous variables. In cases where collinearity was identified, the variable with the greatest correlation with the dependent variable was inputted into the model. All other regression assumptions, including homoscedasticity, linearity, and normally distributed residuals, were also tested. All model assumptions outlined above were within reason, so we proceeded with our regression analysis.

After controlling for age (Table 2, Model 2), multiple linear regression analyses revealed statistically significant associations (*P* < .05) between self-efficacy and: (i) performance accomplishment (health-directed behavior), (ii) verbal persuasion (informational support), and (iii) affective state (depressive symptoms). Vicarious learning did not significantly predict self-efficacy. Overall, this model was statistically significant (*P* < .001) and explained 43% of the self-efficacy variance.

## Discussion

### Summary

Our findings indicate that performance accomplishment, affective state, and verbal persuasion significantly predict health promotion self-efficacy, during times of physical and social distancing, with affective state and verbal persuasion having the greatest association with self-efficacy.

### Limitations

As data was collected cross-sectionally, we are not able to establish causality. Additionally, most participants had attained a post-secondary education and self-identified as Caucasian, which may limit the findings’ generalizability. Furthermore, due to the nature of self-reporting with a trained assessor, outcome measures may have been influenced by social desirability bias. Finally, our small sample of convenience is likely not representative of the broader older adult population in Canada.

### Significance

Our findings provide evidence in support of performance accomplishment, verbal persuasion, and affective states as independent predictors of health promotion self-efficacy in the context of physical and social distancing. In agreement with our findings, studies highlight how performance accomplishment experiences in activities such as physical activity participation can bolster self-efficacy.^23^ In terms of the relationship between affective state and self-efficacy, 1 study on social media users during COVID-19 found that increased emotional arousal can have a significant positive effect on using social media for health information seeking behaviour.^24^ On the other hand, the non-significant relationship between vicarious learning and self-efficacy is interesting, as previous studies highlight how elements of vicarious learning including individual observation and social learning can alter behavioural change and increase positive health promotion behaviours.^25^ It is plausible that due to physical distancing requirements during the COVID-19 pandemic, people may not have had opportunities for being in-person and learning vicariously; thus the reason for it not being a statistically significant predictor of health promotion self-efficacy among older adults during the pandemic. Thus, our findings indicate statistically significant associations between performance accomplishment, verbal persuasion, and affective states, and health promotion self-efficacy during COVID-19. These 3 sources of information could be the focus of interventions to improve health promotion self-efficacy during situations that require quarantining, social isolation, and physical distancing.

**So What?**

**What is Already Known on This Topic?**

While Bandura’s Social Cognitive Theory clearly defines 4 sources of information that contributes to health promotion self-efficacy, there is little information on which variables have the most influence during times of social isolation and physical distancing.

**What Does This Article Add?**

This study examines the relative importance of theoretical social cognitive predictors of self-efficacy during times that limit social opportunities.

**What are the Implications for Health Promotion Practice or Research?**

Performance accomplishment, verbal persuasion, and affective states are statistically significant independent predictors of health promotion self-efficacy during COVID-19. These 3 sources of information could be the focus of interventions to improve health promotion self-efficacy during situations that require quarantining, social isolation, and physical distancing.

## Supplemental Material

Supplemental Material - Social Cognitive Predictors of Health Promotion Self-Efficacy Among Older Adults During the COVID-19 Pandemic Supplemental Material for Social Cognitive Predictors of Health Promotion Self-Efficacy Among Older Adults During the COVID-19 Pandemic by Michelle C. Yang, Gurkaran Singh, and Brodie M. Sakakibara in American Journal of Health Promotion
